# Biologically Inspired Emotional Expressions for Artificial Agents

**DOI:** 10.3389/fpsyg.2018.01191

**Published:** 2018-07-13

**Authors:** Beáta Korcsok, Veronika Konok, György Persa, Tamás Faragó, Mihoko Niitsuma, Ádám Miklósi, Péter Korondi, Péter Baranyi, Márta Gácsi

**Affiliations:** ^1^Department of Mechatronics, Optics and Engineering Informatics, Budapest University of Technology and Economics, Budapest, Hungary; ^2^Department of Ethology, Eötvös Loránd University, Budapest, Hungary; ^3^Institute for Computer Science and Control, Hungarian Academy of Sciences, Budapest, Hungary; ^4^Department of Precision Mechanics, Chuo University, Tokyo, Japan; ^5^MTA-ELTE Comparative Ethology Research Group, Budapest, Hungary; ^6^Department of Telecommunications and Media Informatics, Budapest University of Technology and Economics, Budapest, Hungary

**Keywords:** emotion recognition, artificial agent, human-computer interaction, human-robot interaction, ethological approach, ethorobotics, artificial emotion expression

## Abstract

A special area of human-machine interaction, the expression of emotions gains importance with the continuous development of artificial agents such as social robots or interactive mobile applications. We developed a prototype version of an abstract emotion visualization agent to express five basic emotions and a neutral state. In contrast to well-known symbolic characters (e.g., smileys) these displays follow general biological and ethological rules. We conducted a multiple questionnaire study on the assessment of the displays with Hungarian and Japanese subjects. In most cases participants were successful in recognizing the displayed emotions. Fear and sadness were most easily confused with each other while both the Hungarian and Japanese participants recognized the anger display most correctly. We suggest that the implemented biological approach can be a viable complement to the emotion expressions of some artificial agents, for example mobile devices.

## Introduction

New technologies such as Information and Communication Technologies (ICT) have been on the rise in the past decades alongside with the new emerging field of social robotics.

Artificial agents used in ICT devices could be viewed by users as somewhat living beings, similarly to how children regard social robots differently than strictly inanimate objects (Kahn et al., 2006). A biological approach in the design of these agents' embodiment and behavior could enhance the interaction between humans and the artificial agents.

Emotion expression is an important aspect, which could be used as a means of communication for alerts and notifications (e.g., low battery) but it could also be used to make the device or robot more acceptable to humans and easier to interact with (Hudlicka, 2003). In the field of social robotics emotion expression has notable research interests (Breazeal, 2003). Most studies focus on mimicking human facial expressions (e.g., androids, Becker-Asano and Ishiguro, 2011; virtual faces, Gockley et al., 2004) which can prove difficult at the current level of technology, and could incite the Uncanny-Valley effect (Mori, 1970). Some studies use emoticons displayed on the agent's screen. Emoticons are static pictures or looped short animations used as an addition to textual communication between people (Walther and D'Addario, 2001). There is a great variety of emoticons that are based on the major features of human facial expressions associated with certain emotions (Dresner and Herring, 2010), most frequently the six basic emotions (fear, happiness, sadness, anger, disgust, surprise) described by Ekman and Oster (1979). Although above chance recognition of the basic facial expressions of these emotions has been shown across cultures (Ekman et al., 1987) the success of recognition is far from perfect (Russell, 1994). Research has shown that some aspects of emotion recognition are learned, as the cultural differences in recognition decrease with learning via cultural exposure (Elfenbein and Ambady, 2003) and also children up to 10–12 years of age are less successful in recognition of emotions from facial gestures than adults (Durand et al., 2007). The cross-cultural differences of emotion recognition and expression are of special interest in case of East Asian and Western cultures, due to the different display rules of emotion expressions in collectivistic and individualistic cultures (respectively) (Matsumoto et al., 2008; Safdar et al., 2009). Emoticons derived from facial expressions also have cultural dependence (Park et al., 2013), and they require learning as their recognition becomes better with regular exposure (McDougald et al., 2011). Emoticons are highly human-specific and by using facial expressions as their basis, they are not easily variable or individualized. The use of broader attributes of emotion expression could help to facilitate personalization, which is favorable among users (Blom and Monk, 2003).

If we consider less narrow, that is, less human face specific expressions of inner states, we must address the general rules of emotion expression behaviors in animals, which can be observed across multiple taxa (Darwin, 1872; Plutchik, 2001). Defining these rules can give us a set of guidelines that can be used to implement emotion expression systems that are dynamic, can be used across multiple modalities and do not rely on human facial expressions, thus their recognition might be consistent across cultures. In social robotics certain behaviors of social animals are considered as good models for designing functionally analogous behaviors for robots (Miklósi and Gácsi, 2012). Gácsi et al. (2016) used the emotion expression of dogs (*Canis familiaris*) as a basis for the emotionally expressive behavior of a mechanoid-looking robot. Their results show that participants tended to interpret the robot's actions as expressions of emotions and could identify the emotions significantly above chance level.

We propose a similar approach for creating generalized simple expressive behaviors that are based on common biological rules of emotion expressions of multiple animal species.

Animals express numerous behavioral elements in certain situations that are assumed to reflect their inner (emotional) states. Ethological research has revealed some common behavior patterns that are recognizable across wide range of taxa. Next, we summarize some of these observable behaviors and the derived rules that we use for creating our generalized model for displaying emotional states (Table 1). In this study we focused only on the visual displays of animals, but the inclusion of other modalities (e.g., acoustic signals) into the emotion expression displays is also possible.

**Table 1:** Summary of biological rules used for developing the artificial emotion expressions.

**Emotion**	**Function**	**Action**	**Movement and rotation**	**Size**	**Color**
Surprise	Startle reaction	Freezing, orienting at stimulus	Normal rotation stops/freezes. Grid rotates 90° in the opposite direction. Rotation stops again.	Sphere and grid increase slightly	Sphere and grid becomes slightly brighter
	Barto et al., 2013	Sokolov, 1963; Plutchik, 1980; Scherer, 2001	Sokolov, 1963; Plutchik, 1980; Yamashita et al., 2000	Lucey et al., 2010; Descovich et al., 2017	Kim et al., 2004
Fear	Avoidance, escape	Pale colors, size decrease, seeking escape, hiding	Agent moves toward the right upper corner. Rotation speed increases.	Sphere and grid size decrease, sphere becomes bigger than grid	Sphere becomes pale/bright
	Bonenfant and Kramer, 1996; Stankowich and Blumstein, 2005	Dill and Houtman, 1989; Bonenfant and Kramer, 1996; Stankowich and Blumstein, 2005	Dill and Houtman, 1989; Bonenfant and Kramer, 1996; Stankowich and Blumstein, 2005; Rhoades and Blumstein, 2007	Caro, 2005	Conte, 2004; Vianna and Carrive, 2005; Garfinkel and Critchley, 2014
Anger	Approach/avoidance, intra-specific competition (enemy)	Vivid colors, size increase, threat displays (showing weaponry)	Rotation speed increases. Agent moves closer (size increase).	Sphere and grid size increase, grid becomes bigger than sphere	Sphere becomes red, grid becomes blue
	Scott and Fredericson, 1951; Evans and Norris, 1996	Evans and Norris, 1996; de Boer et al., 2003	Dill, 1978; Nishida et al., 1999; de Boer et al., 2003	Scott and Fredericson, 1951; Evans and Norris, 1996; Nishida et al., 1999; de Boer et al., 2003	Evans and Norris, 1996; Drummond and Quah, 2001; Chen and Fernald, 2011
Happiness	Approach to contact, reach goal, play	Seeking proximity, jumping motion, hiding weaponry, vivid colors	Agent moves closer (size increase) and downwards, bounces on the bottom. Grid keeps rotating.	Sphere and grid size increase, sphere becomes bigger than grid	Sphere becomes orange, grid fades
	Panksepp, 2004; Trezza et al., 2010; Konok et al., 2015	Izikson et al., 2006; Trezza et al., 2010; Konok et al., 2015	Pellis and Pellis, 1983; Pellegrini and Smith, 1998; Cordoni, 2009; Held and Špinka, 2011	Topál et al., 2005; De Marco et al., 2014; Konok et al., 2015	Izikson et al., 2006
Sadness	Contact/care seeking	Slow motion, pale colors, size decrease	Agent moves slowly to the side. Rotation speed decreases considerably.	Sphere and grid size decrease slightly, grid becomes smaller than sphere	Sphere fades, becomes transparent
	Plutchik, 2001	Herrera-Pérez et al., 2008; Nestler and Hyman, 2010	Panksepp et al., 1991; Michalak et al., 2009; Buyukdura et al., 2011; Konok et al., 2015	Panksepp et al., 1991; Michalak et al., 2009; Buyukdura et al., 2011; Konok et al., 2015	Conte, 2004; Fitze et al., 2009
Neutral	No specific function	Normal operation of the agent	G rotates at an intermediate, constant speed	Sphere and grid remain their original size	Sphere is green, grid changes randomly

*The table is partially based on Plutchik (2001)*.

We chose the basic emotions that have an ecological function and can be relevant in social robots or mobile agents: anger, happiness, fear, surprise and sadness. These emotions can be interpreted as coordinates in a dimensional model, e.g., the circumplex model of affect by Russell (1980) in which emotions are positioned based on their valence (positive or negative) and intensity (high or low). In the following summary the described emotions are listed based on their intensity (Russell, 1980).

### Surprise

It is assumed that the reaction to sudden changes in the environment is caused by a mismatch between the observed and the expected environment or event, and is accompanied by a change in the emotional state referred to as surprise (Barto et al., 2013). It is a relatively short emotional display and can involve momentary freezing (staying motionless) (Plutchik, 1980), orientation at the source of stimulus (Sokolov, 1963) or a combination of them. Novel stimuli elicit alertness (Scherer, 2001), which can be accompanied by the widening of eyelid apertures (Descovich et al., 2017), by increased visibility of the white sclera (Kim et al., 2004), and by raised eyebrows (in humans: Lucey et al., 2010). Surprise in itself cannot be categorized as having a positive or negative valence as it is based on the circumstances (e.g., if the stimulus is a suddenly appearing conspecific or a predator) therefore it is difficult to generalize a rule for the avoidance or approach behavior. The startle reaction usually contains stereotypic behaviors, for example in certain tadpoles the sudden stimulus causes them to quickly bend their tails and move forward in a different direction than before (Yamashita et al., 2000). Thus, for the creation of the surprise emotion the virtual agent displayed freezing, change of orientation, and an alert state with a slight size increase and brightened color.

### Fear

Behavioral reactions associated with fear can be observed in animals e.g., during predator-avoidance and retreating, frequently characterized by the flight initiation distance (Stankowich and Blumstein, 2005). The animals might have the potential for active avoidance which results in fast locomotion and fleeing from the approaching predator (Bonenfant and Kramer, 1996). The goal of the fleeing is usually to gain distance from the predator and to reach refuge (Dill and Houtman, 1989) where they can hide (Rhoades and Blumstein, 2007) which is often helped by a crouching posture (Caro, 2005). As fear is often the result of a stressful stimulus, pale colors can also occur (Conte, 2004; pallor in humans: Garfinkel and Critchley, 2014) due to the redirection of blood from the skin and periphery to the muscles (Vianna and Carrive, 2005). In the fear display the agent flees quickly to the corner of the screen, its size decreases and it displays paler colors.

### Anger

The majority of threat displays follow general rules, as many evolved from movements and behaviors that occurred before or during fighting (Andersson, 1980). Agonistic behaviors contain threat displays, the display of weaponry, size exaggeration (Nishida et al., 1999; de Boer et al., 2003), vivid colors e.g., via blood rush (Drummond and Quah, 2001), or showing vividly pigmented areas during aggressive displays (Evans and Norris, 1996; Chen and Fernald, 2011), and typical approach patterns (Scott and Fredericson, 1951). The approach patterns can be part of ritualistic behaviors intended to deal with the conflict without an actual fight, or could be the general dynamics of an aggressive approach (starting with orientation and a slow approach, followed by a fast movement to reach the target) (Dill, 1978). Thus, for the creation of the anger display the virtual agent displayed vivid color, accelerating approach, increase in size, and angular forms (display of “weaponry,” similarly to teeth or talons).

### Happiness

This emotion was based on the play and greeting behavior of animals which are both connected to positive emotional states (Panksepp, 2004; Trezza et al., 2010; Konok et al., 2015). During play animals exhibit a wide range of behavior elements that can originate from other contexts, e.g., from fighting or sexual behaviors (Palagi et al., 2016) which we did not want to include, but less specific behaviors, e.g., contact seeking, locomotor-rotational and jumping movements are found in both animals and humans (Pellis and Pellis, 1983; children: Pellegrini and Smith, 1998; Cordoni, 2009; Held and Špinka, 2011) while increased blood flow due to physical activity can result in facial flushing in humans (Izikson et al., 2006). The greeting behavior of dogs is interpreted as happy or joyful by humans (Konok et al., 2015), however such behavior can be also observed in socialized wolves (Topál et al., 2005), and macaques (De Marco et al., 2014). Many aspects of this behavior can be considered an opposition to agonistic behaviors, although fast movements are characteristic in both instances. Thus, for the creation of the happy display the virtual agent displayed fast and energetic movements, jumping, approach to contact and less angular forms (hiding “weaponry”).

### Sadness

In animals behavioral changes caused by stress which the animal cannot cope with may evoke anhedonia, and a passive, withdrawn behavioral state (Herrera-Pérez et al., 2008; Nestler and Hyman, 2010). In some species there are characteristic changes in coloration due to elevated stress, as the color of the animal fades e.g., in fish (Conte, 2004) and in case of chronic stress, reptiles (Fitze et al., 2009). Depression and chronic sadness is usually associated with slow movements and contraction of the body/hunched posture (Panksepp et al., 1991; Michalak et al., 2009; humans: Buyukdura et al., 2011; Konok et al., 2015), resulting in smaller perceived body size. Thus, for the creation of the sadness emotion expression the virtual agent displayed decreased size, fading coloration and slow movements.

We tested this new approach by developing emotion displays of basic emotions (happiness, anger, fear, surprise, sadness) and a neutral state for an artificial agent by dynamically changing its visual attributes. The attributed changes follow general biological rules that are based on animal behavior and communication of inner states. A set of published studies that use abstract forms for complex emotion expression in ICT devices describe the eMOTO (Fagerberg et al., 2003, 2004; Sundström et al., 2005), which is designed to enrich text messages with affective content. The system expresses emotions by using dynamic color and shape background themes of the text message application via changing its color, texture and the movement of forms constituting the texture. The creation of the expressions seems to draw from an artistic approach, as the reasoning behind the expression design is not discussed in detail and not well-referenced. Color changes are also used in other studies to express emotions in social robots e.g., with specific light patterns evoking rain or fire (Feldmaier et al., 2016), dynamic changes of luminosity and hue (Terada et al., 2012), or in a multimodal system together with vibration and sound signals (Song and Yamada, 2017), but the designated features of the emotion expressions are not based on biological rules or are set by the participants as part of the experiment.

In our study we used a complex, abstract form as the structure of our agent as we wanted to emphasize the being-like complexity of the agent while avoiding similarities with any existing organisms or symbols. Our aim was to create an agent that can display emotions in short, few seconds long animations. As this agent provides only one possible model for the proposed approach, we did not offer several variations of it or search for the very best displays, but only wanted to demonstrate that a more diverse set of emotion displays can be developed using dynamic changes in the agent's “body” movements, appearance and “approach-avoidance” behaviors. We used the visual modality as a basis, but other modalities (e.g., auditory, tactile) could be integrated in the future.

The artificial agent (originally named EDA: Emotion Display Agent) was developed by György Persa and Péter Baranyi using Ogre3D graphics rendering engine. The display animations were recorded with Fraps®. The user interface of the agent enabled the researchers to change multiple parameters of its embodiment, movement pattern and dynamic changes (Baranyi et al., 2011; Persa et al., 2012). Table 2 contains the description of changeable parameters.

**Table 2:** Adjustable parameters of the artificial agent.

**Parameters**	**Description**
Implementation time	The speed at which a modified parameter changes the agent display (sec)
Sphere color	Sets the color of the sphere (RGB values)
Transparency of sphere	sets the transparency of the sphere on a scale from 0 to 1 (from totally transparent to opaque)
Sphere size	Sets the size of the sphere compared to the original size (scale, from no sphere → to sphere filling out the screen)
Distance	Sets the direction of movement of the agent and its size/distance (vector)
Grid rotation speed	Sets the rotation speed of the grid (1/s)
Grid rotation axis	Sets the rotation axis (3d vector)
Grid rotation distortion	Sets the measure of rotation asymmetry
Grid size	Sets the size of the grid independently from the size of the sphere (scale)

**Figure 1:**
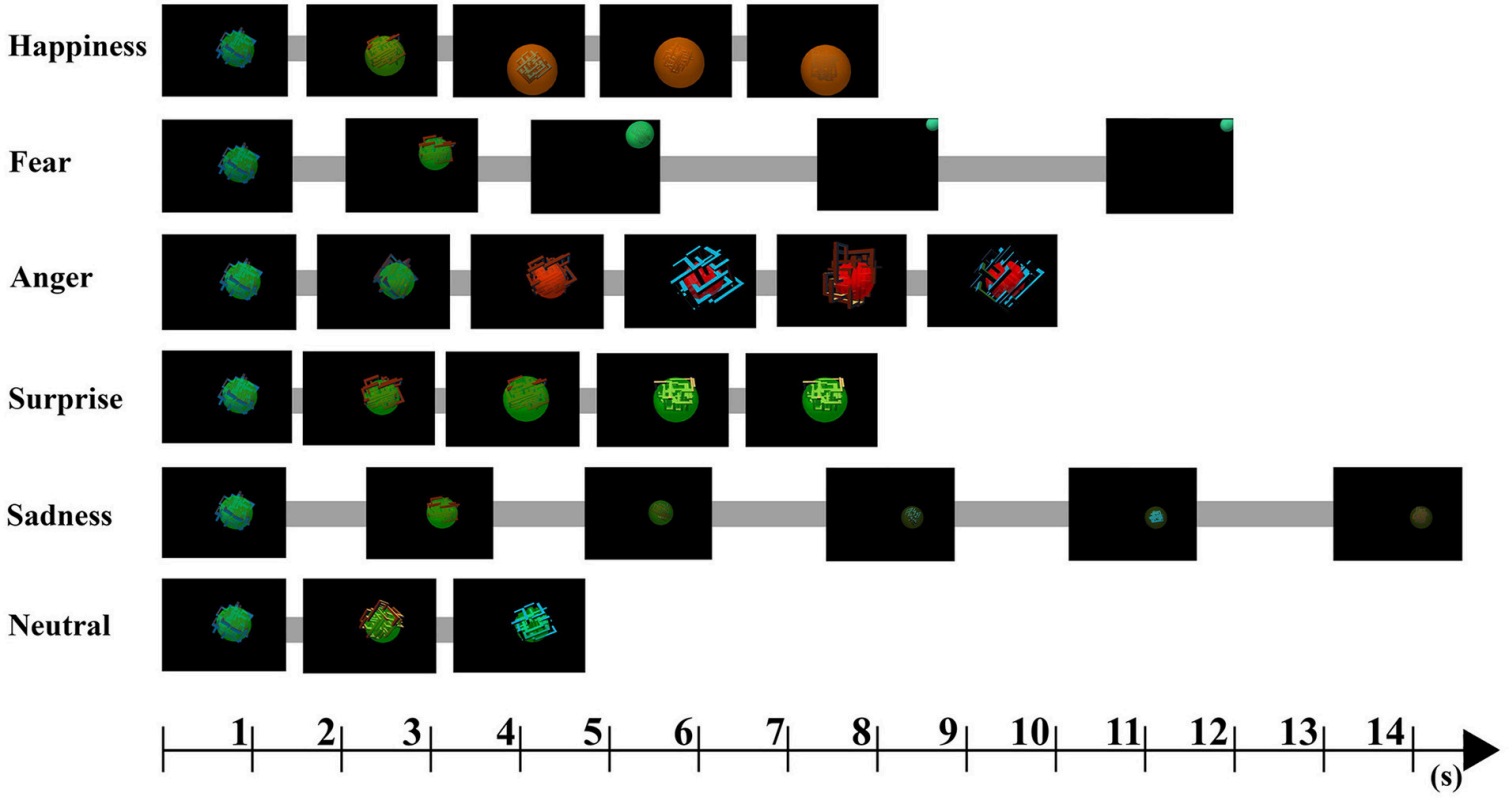
The figure shows the dynamic changes of the agent during the emotion displays. The starting state is the same for all displays (happiness, fear, anger, surprise, sadness, neutral).

The agent is comprised of a sphere and a labyrinth-like, angular part, “grid” (Figure 1). This structure was chosen to accommodate some visual properties that could make the agent look more similar to a living being. The combined movement and relative size change of the sphere and the grid enables the agent to be seen as rounder or more angular, depending on the displayed emotion.

The agent is shown on a screen and can visualize basic emotions in 2D, but due to its form and movements, it is generally perceived as a 3D object/being. The emotions expressed by the agent were designed to be displayed in a given time limit between 4 and 15 s. The differences in the display durations reflect some of the potential variability found in the temporal dynamics of emotions and emotionally expressive behaviors (facial expressions: Ekman, 1984; Frijda et al., 1991; Verduyn et al., 2015), and are also the result of specific behaviors (e.g., hiding increases the length of the fear display).

Hypothesis

1. We expected that participants would recognize the generalized simple expressive behaviors giving the highest score to the respective emotions in multiple choice questions.
2. We also expected lower recognition rates for simple emotional states that share more behavioral attributes.
3. We also hypothesized that our displays present universal biological features, thus there would be no major differences in the assessment of the Hungarian and Japanese participants.

## Methods

### Subjects

Both the Hungarian and Japanese subjects were unpaid volunteers recruited from university students. The Hungarian sample consisted of 114 participants, 78 women and 34 men (age 21 ± SD 1.7 years). The Japanese sample consisted of 22 participants, 21 men and one woman (age 22 ± SD 1.1 years). The Hungarian sample, due to the higher number of participants, was tested in six groups. For the cultural comparison a balanced Hungarian sample was used.

The study was carried out in accordance with the recommendations of the Institutional Review Board of Institute of Biology, Eötvös Loránd University, Budapest, Hungary. The protocol was reviewed and approved by the Institutional Review Board of Institute of Biology, Eötvös Loránd University, Budapest, Hungary. All subjects gave informed oral consent in accordance with the Declaration of Helsinki. The study was conducted in 2011, when written consent was not required for questionnaire studies. Participants took part in the study voluntarily and anonymously.

### Procedure

The testing took place at the Department of Ethology at the Eötvös Loránd University in Budapest, Hungary and in the Human-System Laboratory at Chuo University in Tokyo, Japan.

The emotion displays were defined for each emotion based on the above ethological summary and were recorded as short videos.

#### Preliminary study

We used human fine tuning as a second step to finalize the design of the emotion expressions: we conducted a single questionnaire preliminary study with this first set of animations to get a pre-evaluation of the displayed emotions. The participants of the preliminary study were Hungarian volunteers of the staff and students of the Department of Ethology at the Eötvös Loránd University (*N* = 13, 8 females and 5 males, age 34.4 ± SD 10.2 years). The experimenter used a Microsoft PowerPoint® presentation to play the 5 videos in a fixed order (anger, fear, happiness, sadness, and surprise), the experimenter changed the slides manually after each video.

Before viewing the videos, the experimenter told the subjects to consider the agent as an unknown being and that the subjects should fill out the closed-ended questionnaire according to what emotions they think the agent expresses, which emotion is more adequate for the displayed animation.

The instructions for the questionnaire were: “Mark for each emotion how much it is relevant for the agent (‘living being’) on the given video (to what extent it describes its actual inner state).” After the subjects watched the videos, the experimenter handed out the questionnaire. The subjects then viewed the animations again one by one and the subjects were instructed to fill out the relevant parts of the questionnaire after each video. The subjects had to mark on a Likert scale (from 0 to 5) how characteristic each emotion (angry, happy, afraid, surprised, sad) was to each video. The questionnaire data of the preliminary study were analyzed with simple sum of scores for each emotion from all the participants.

The results of the preliminary study showed that the summed scores for most emotions were the highest in case of the relevant videos, e.g., when the subjects viewed the video in which the agent displayed sadness, the subjects gave the highest score to the sad emotion. The only exception was the happiness display, in which the participants gave slightly more scores to the anger emotion (happy 36, angry 41 scores).

#### Finalized procedure

Based on the results of the preliminary study and on the interviews conducted with the participants, some changes were made in the displays, and we also added a neutral state display in order to have a sort of “baseline.” The finalized attributes of each display are summed up in Table 1, while the dynamic changes of the agent during the emotion displays can be seen in Figure 1. The videos are available in the supplementary material. All displays started the same way, showing the agent in its neutral state (greenish sphere color, visible medium sized grid). Based on the results of the preliminary study, the questionnaire was divided into two parts, in Q1 we used open-ended questions in which the participants could freely express what they thought the displays conveyed. The instruction on Questionnaire 1 was: “Let's suppose that the agent on the video is an unfamiliar living being. What do you think its current thought/mood is, how do you think it is feeling right now? (not sentences, just words!)”. In Questionnaire 2 we used the same closed-ended questions as in the preliminary study. We analyzed the two questionnaires separately.

Due to the two questionnaires the process of the presentation was modified. There were three presentations. In the first presentation the videos were all played in a single succession (watching video1-video6 in random order that differed for the 6 Hungarian groups of participants). Next, the participants received Q1 with the open-ended questions and the videos were presented to them again but this time one by one, in a random order that differed from the order of the first viewing. The participants were instructed to fill out the relevant part of Q1 after each video. Then Q1 forms were collected, and Q2 questionnaires with the closed-ended questions were given to the participants. The subjects then were presented with the videos the third time, in another random order and were instructed to fill out Q2. The random orders for the videos were defined a priori, and the participants were identified through a codename. The test procedure was the same in both countries, but in case of the Japanese group although the open-ended questionnaire was filled out by participants as in the Hungarian group, it was later discarded from analysis due to difficulties in translating the responses into terms that properly correspond to the Hungarian ones. In case of the Japanese participants, the random order of viewing was the same as one of the Hungarian group's.

### Data analysis

#### Open questions (Q1)

The answers for each video (separately for each viewing) were collected and categorized. If the participant used multiple definitions/adjectives, only the first three were considered. We used a scoring system in a modified form, adapted from another study on emotion expression in robots (Gácsi et al., 2016) to categorize the given answers. Answers with scores 1 or 2 were words that referred to not being-like objects (that could still show behaviors and perform actions, e.g., machines). Answers with scores 3 or 4 related to concepts that could be associated with the inner states or emotions of living beings.

Score 1: formal description of the observed behavior (e.g., agent changes color, spins faster).

Score 2: indicating some contextual behavior that attributes a meaning to the video which cannot be directly observed from the behavior of the agent (e.g., learns something, runs).

Score 3: mentioning a term or phrase, which implicitly indicates some inner state but without naming that concrete emotion (e.g., threatens somebody, tries to escape, reclusive).

Score 4: naming an inner state or emotion (or the lack of emotion) explicitly either as a verb, a noun or an adjective (e.g., happy, it is afraid of something, neutral, tired). We used descriptive analysis to show the ratio of types of answers based on the scoring system. The answers that received scores 3 or 4 were analyzed further. In the next step the answers that received scores 3 or 4 were selected and each expression was counted. We created answer groups based on the expressions that occurred more than five times per video, as expressions with similar meanings were grouped together, resulting in 5–8 categories per display.

#### Closed questions (Q2)

In the Q2 questionnaire the participants had to mark on a Likert scale from 0 to 5 for each emotion their relevancy to the emotion displayed in the given video, therefore the participants did not only choose one emotion per video.

#### Descriptive analysis

We measured success of recognition by giving 1 point if participants gave the biggest score to the correct emotion and 0 if they gave it to another (in case the participant gave the same high scores to other emotions as well as to the correct one, we divided the 1 point by the number of emotions that got the same score). We also measured if the participants gave the maximum 5 score to the correct emotion. Next, we summed the scores of the emotions separately in case of each displays (e.g., summed the scores given by all participants to the happy emotion in the neutral display) to show how all the emotions were scored for each display. Finally, we created confusion matrices to show which emotions were mixed up by the participants, by counting the percentage of answers that gave the biggest score in case of each emotion, similarly to measuring success (the score given for an emotion received 1 point if participants gave the biggest score to that emotion; in case the participant gave the same points to other emotions as well, we divided the 1 point by the number of emotions that got the same score).

#### Statistical analysis

Statistical analysis was conducted with IBM® SPSS® Statistics 20. As a first step, we compared the success of recognition in each display to chance level (binomial test with 0.2 as chance level). To obtain dichotomous data we used two derived variables for measuring success to chance level, by creating both a permissive (largest value given for the correct emotion, the same score could also be given to other emotions) and a strict (only the correct emotion received the largest score) success variable, e.g., if the Sadness display received a score 4 for both the sad and angry emotions, it was considered successful by the permissive, and unsuccessful by the strict success variable.

Next, we used Friedman tests and Wilcoxon signed rank tests, with Benjamini-Hochberg corrections to examine whether the scores given to the correct emotions differed significantly in the displays from the scores given to the other emotions. We conducted a hierarchical cluster analysis to examine the closeness of scored emotions (cluster method: Ward's method; variables: scored emotions; counts: chi-squared measure). Finally, we used Generalized Linear Mixed Models (GLMM) with the fixed effects of the participants' gender, the category of the scored emotion (happy, angry, afraid etc. emotions), the type of display (Happiness, Anger etc. videos), and their two-way interactions. The targets were the scores given for all the emotions across all displays and the random effects were the subjects. We used multinomial probability distribution and cumulative logit link function.

## Results

### Hungarian sample

#### Open questions (Q1)

**Figure 2:**
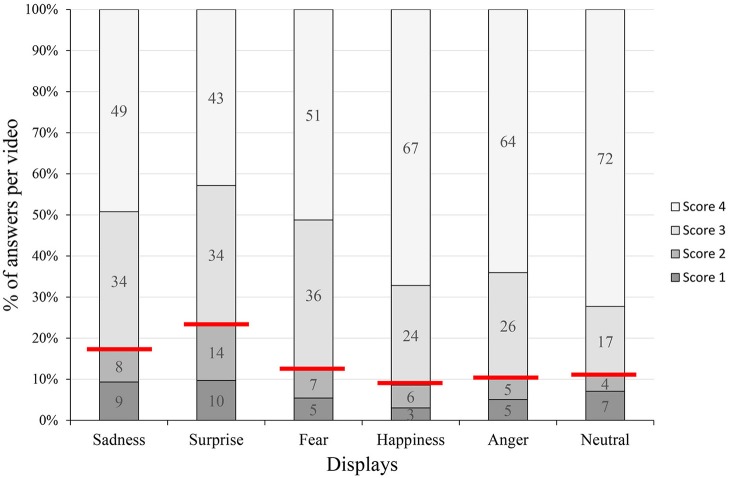
The figure shows the percentage of answers given to the open-ended questionnaire in terms of the types of answers. The red lines indicate the separation of scores 1–2, indicated by dark gray colors (words referring to not being-like agents) and scores 3–4, indicated with light gray (words referring to being-like agents). Answers receiving scores 3 and 4 were analyzed further. Score 4: naming an inner state or emotion (or the lack of emotion) explicitly; score 3: mentioning a term or phrase, which implicitly indicates some inner state but without naming a concrete emotion; score 2: indicating some contextual behavior that attributes a meaning to the video which cannot be directly observed; score 1: formal description of the observed behavior.

As the first step of the descriptive analysis we summarized the answers based on the scoring system. In all displays the proportion of scores 3 and 4 were high. The answers that received scores 4 or 3 amounted to at least 77% of all answers (in case of the Surprise display) and at most 91% (in Happiness), meaning that the vast majority of respondents used words that express or imply emotions or inner states that are characteristic of living beings. The Happiness, Anger and Neutral displays prompted answers that were direct expressions of emotions (score 4) in 64–72% of the cases (Figure 2). Answers receiving scores 3 and 4 were further analyzed. Inter-observer reliability for separating answers with scores 1–2 from the ones with scores 3–4 was assessed by two observers on a balanced sample using Cohen's Kappa (*k* = 0.7; *p* < 0.001). Cohen's Kappa is a frequently used method for measuring inter-observer reliability, where *k* = 0.7 is considered a substantial agreement (Viera and Garrett, 2005).

Most answers for the Sadness display were distributed between the “sad, lonely” (17%), the “tired, resting” (17%), “shy, reclusive” (16%) and “calm” (13%) categories, while a smaller number of expressions belonged to the “afraid, hides” (8%) and thinking (6%) groups. The “sad, lonely” category had one of the highest percentages, but there were many expressions connected to inner states such as tired, sleepy, thinking. The words used to describe the Surprise display also covered a big array. Most answers were in the correct “surprised, confused” category (16%), while the meaning of some other groups could be also related to surprise, such as “curious, inquiring” (8%), or “nervous, startled” (6%). On the other hand, some answers either depicted the display as “happy, satisfied” (11%), “neutral, indifferent” (10%), “confident” (7%), “thinking” (6%), or “calm” (4%).

**Figure 3:**
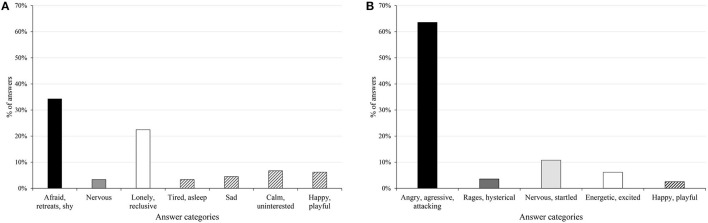
The categories of answers given for the Fear **(A)** and Anger **(B)** displays. The darker shades signify the correctness of the answers, the white column shows the answers that are neutral in the case of the emotion at hand, while the striped columns indicate somewhat opposing meanings.

The Fear display was correctly recognized in 34% of the cases in which the used words belonged to the “afraid, retreats, shy,” and 3% to the related “nervous” category (Figure 3A). The second largest group was the “lonely, reclusive” (22%), followed by the opposing “calm” (7%), “happy” (6%), “sad” (4%) and finally the “tired” (3%) categories. The Happiness display yielded less categories, with the leading “happy, playful” at 28%, followed by groups that all convey a similarly high arousal level: “energetic, excited” (14%), “nervous, stressed” (9%), curious (3%). A high percentage of answers fall into the “angry, aggressive” group, which has a directly opposite meaning in valence, but the same level in arousal. The clearest division can be seen in the case of the Anger display (Figure 3B), in which 64% of the participants answered with words that are in the correct “angry, aggressive, attacking” category. It is followed by “nervous, startled” (11%), “energetic, excited” (6%) and “rages, hysterical” (4%). The answers conveying emotions with opposite valence but similar arousal level are also present in smaller numbers in the “happy, playful” (3%) group.

Finally, in the Neutral display most of the answers corresponded to the intended inner state. 32% gave answers that fit into the “calm, balanced,” and 19% into the “neutral, indifferent” category. 9% attributed positive valence to the display (“happy, good mood”), while the rest of the answers fell into the “tired, resting” (7%), “bored” (4%) and “thinking” (4%) groups.

#### Closed questions (Q2)

##### Descriptive analysis

**Figure 4:**
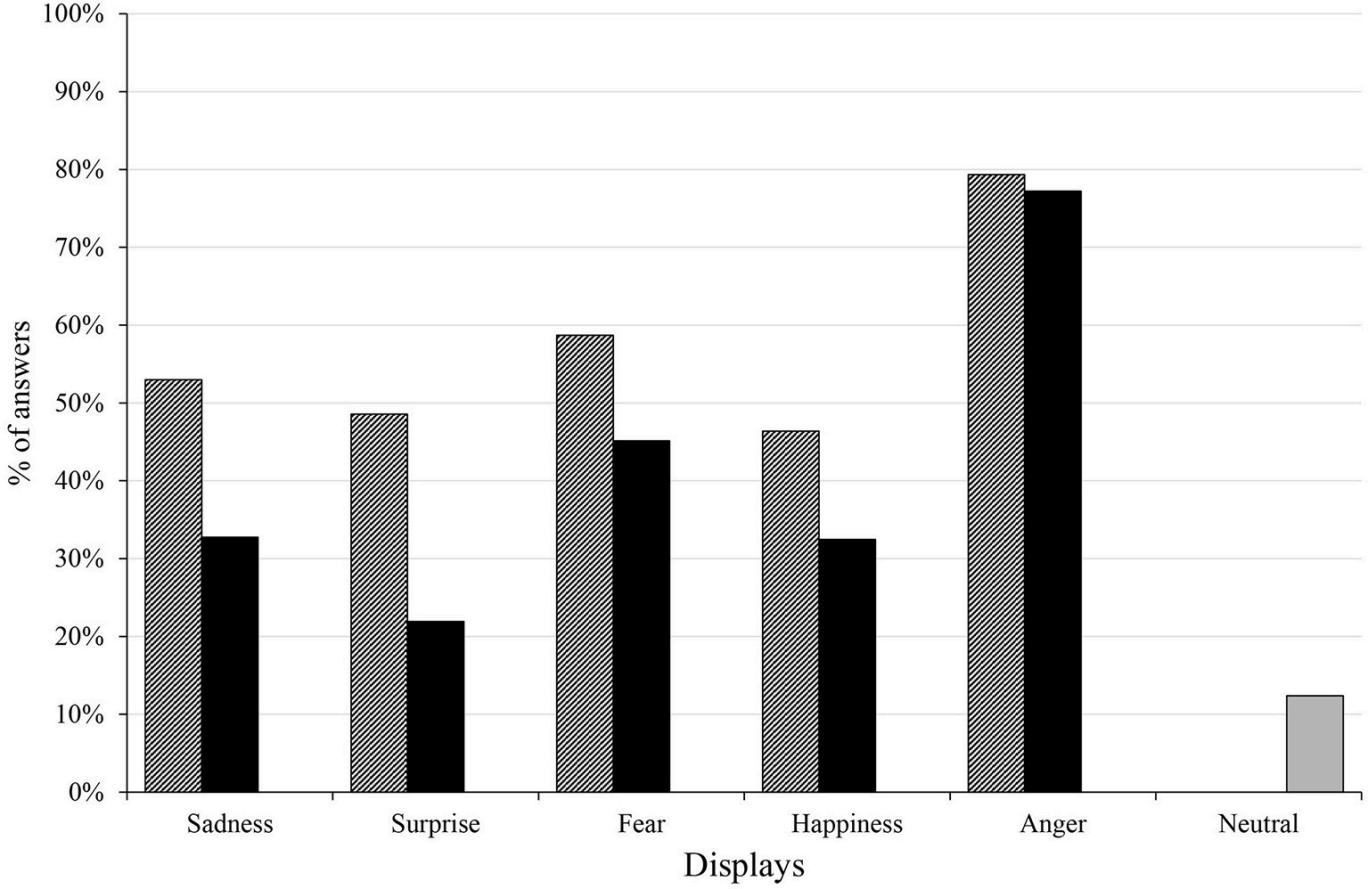
The striped columns show the largest score given to the correct emotions (in case the same high scores were given to other emotions as well as to the correct one, the 1 point was divided by the number of emotions that got the same score), the black columns show the maximum 5 score given for the correct emotions, while the gray column depicts the percentage of answers in which the participants gave 5 scores for any emotions in case of the neutral display in the Hungarian group.

We demonstrate the success of recognition in Figure 4, with two different depictions of success: the percentage ratio of the highest scores given to the correct emotion and the percentage of answers that gave the maximum 5 scores to the correct emotion.

In three displays more than half of the participants gave the largest score for the emotions that the agent was meant to display, while in the Surprise display 49%, in the Happiness display 46% of them gave the highest score to the right emotion. Similarly to the open questions, anger proved to be the most evident display, as 79% of the participants gave the highest score to the correct emotion, which was almost always the maximum 5 score. The differences between the answers that gave the largest value to the right emotion, and the ones that gave the maximum 5 score indicate the certainty with which the participants chose the correct emotion. In case of the surprise display, despite of almost half of the participants gave the largest score to the right emotion, it was the maximum 5 score only in 22% of the answers.

The success of the neutral state display is visible by the small number of answers (merely more than 10%) who gave the maximum 5 points to any emotions when viewing the neutral state video, indicating that the participants did not strongly associate the neutral state with any of the five emotions.

**Figure 5:**
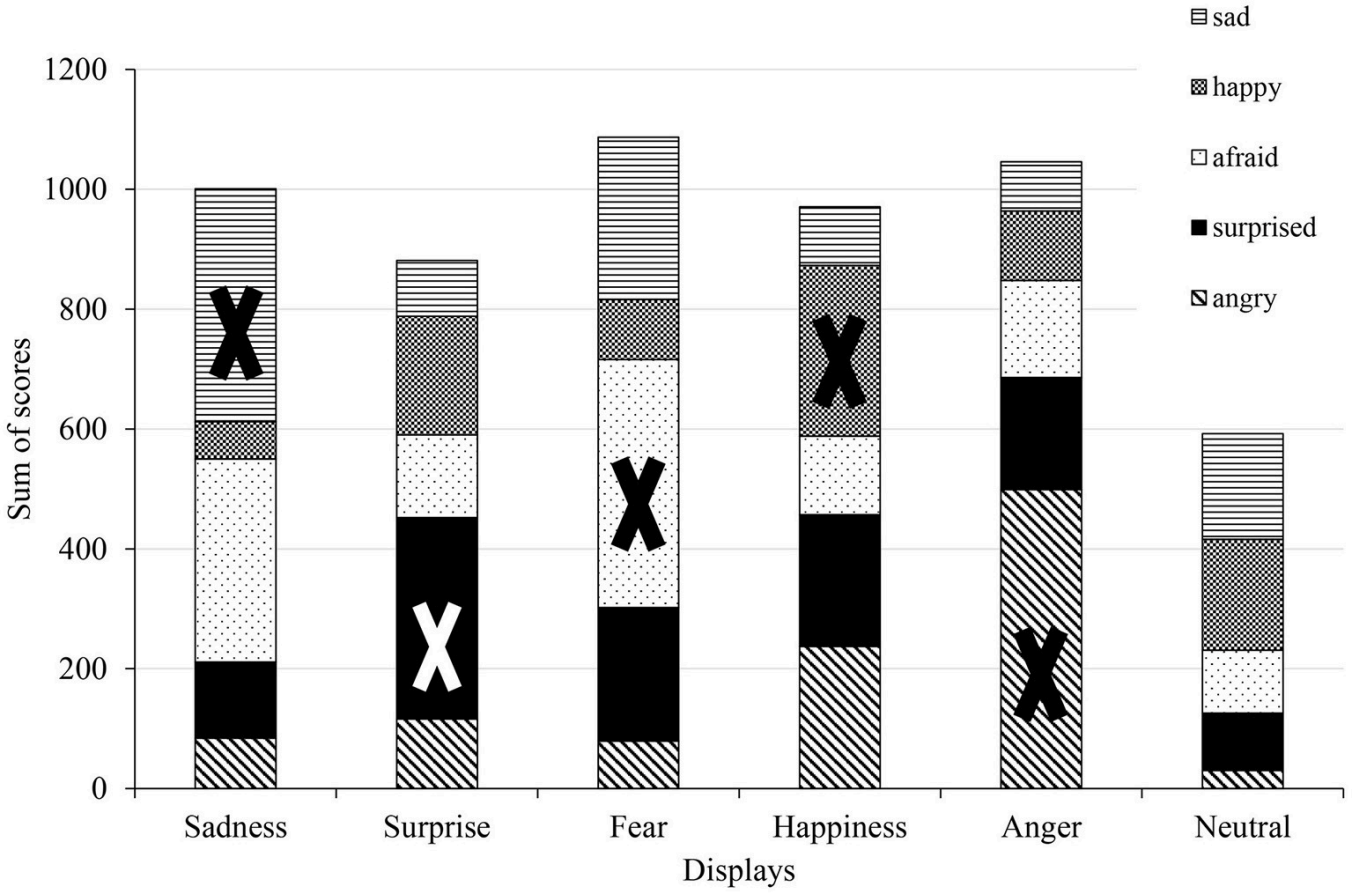
Analysis of sum of scores in the Hungarian group. The X sign shows the correct emotions in each display.

The analysis of sum of scores (Figure 5) shows that Anger is once again the most clearly recognized display, and that the neutral state received generally low scores. The closest scores are present at the Sadness display (sad: 389; afraid: 339 scores) and in the case of Happiness, where the participants also gave high scores to anger (happy: 285; angry: 237 scores).

**Figure 6:**
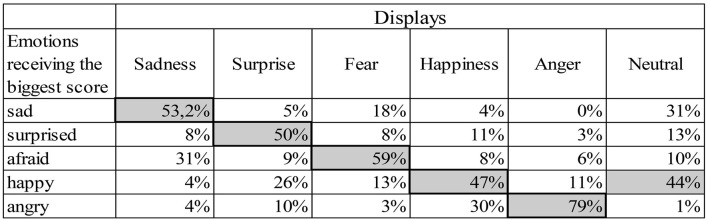
Confusion matrix of answers in the Hungarian group. The gray cells indicate the percentage at which each emotion received the biggest score. The correct emotions are indicated by dark borders.

We created a confusion matrix, based on which emotion received the biggest score from the participants in each display. Figure 6 depicts which emotions were mixed up more often by the participants. In the Sadness display the fear emotion was typically mixed with the sad, and in the Happiness display angry was confused with the happy emotion. The neutral display was generally considered to show happiness or sadness.

##### Statistical analysis

The recognition of the displayed emotions was significantly higher than chance level in both the permissive and strict dichotomous success variables (permissive: all *p* < 0.001; strict: all *p* < 0.001).

**Figure 7:**
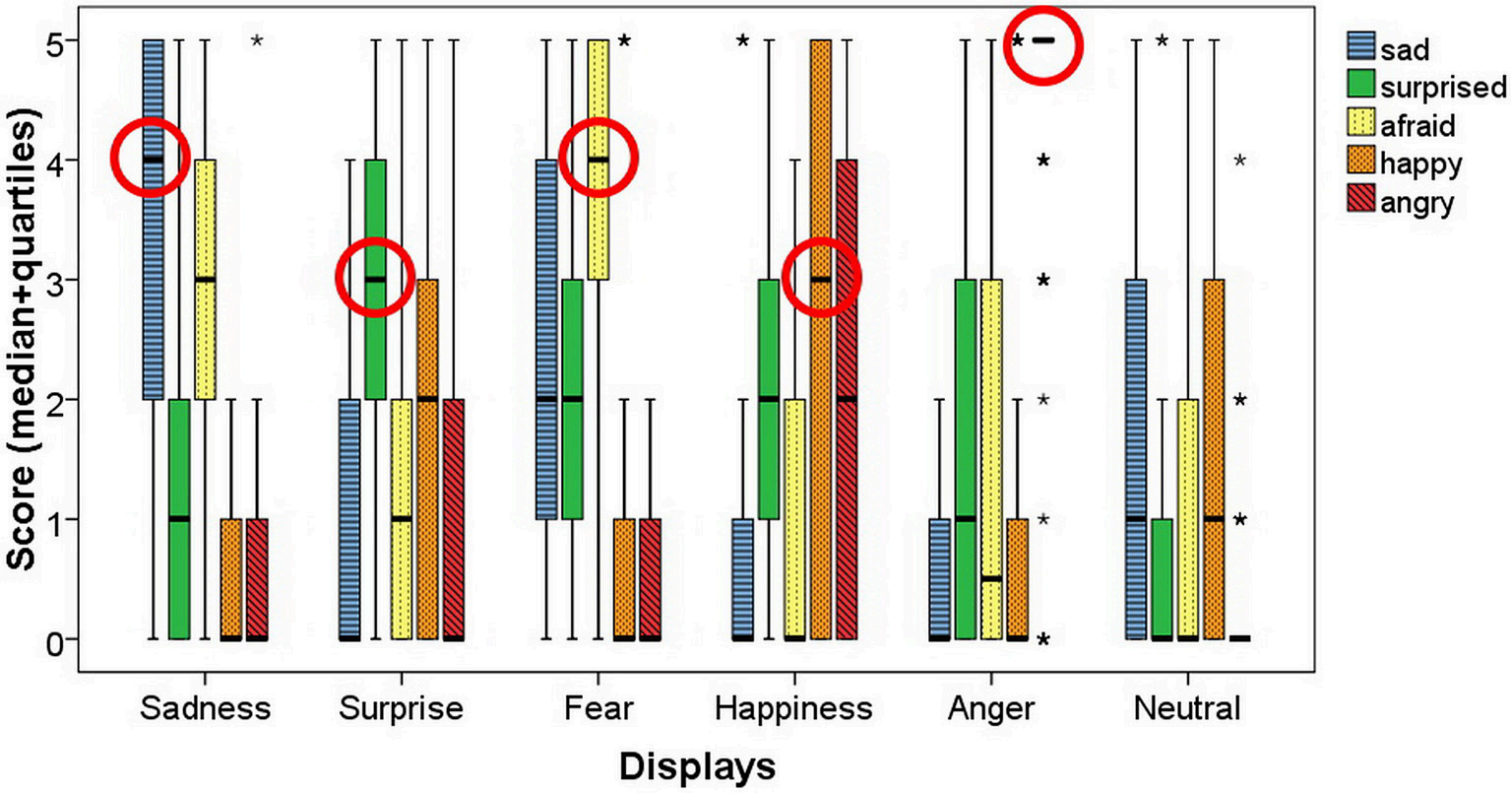
The figure shows the scores given for the six displays, while the circles show the medians for the scores given to the correct emotions by the Hungarian participants. The * indicates outliers.

**Figure 8:**
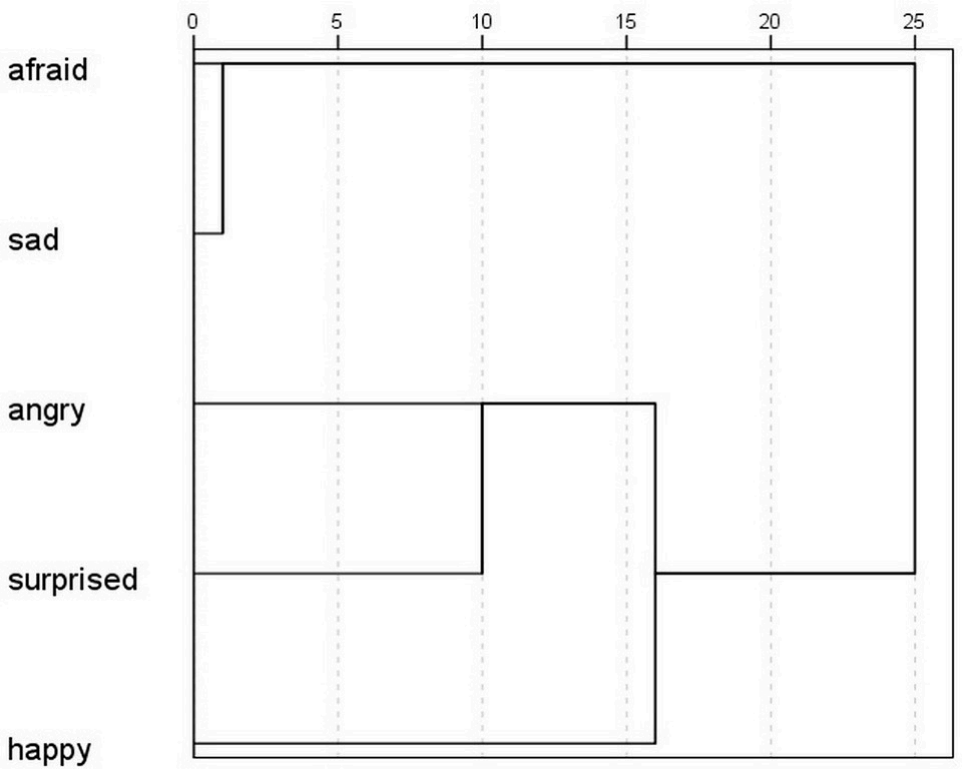
Ward's hierarchical cluster analysis, showing the similarities of scores given to each emotion by the Hungarian participants.

The scores given to the correct emotions were significantly higher than the scores given to all the other emotions in four displays (Fear, Surprise, Sadness and Anger) (Friedman test + Wilcoxon signed rank test; ^***^p < 0.001, except in the Sadness display, where the difference between the correct answer (sad) and the scores of the afraid emotion was significant at *p* = 0.042). In the Happiness display the scores of happy and angry were not significantly different (*p* = 0.116) (Figure 7). The cluster analysis showed which emotions were perceived by participants as occurring together (Figure 8). Based on the results, the afraid and sad emotions had similar score patterns across displays, while the scores for happy, angry and surprise emotions were closer together. In the GLMM analysis the overall corrected model was significant [*F*_(59, 3281)_ = 18.216; *p* < 0.001; *n* = 3345]. The participants' gender had no effect on the scores, and neither did the gender–display type and gender–emotion category interactions. The effects of display type [*F*_(5, 3281)_ = 20.554; *p* < 0.001; *n* = 3345] and emotion category were significant [*F*_(4, 3281)_ = 9.823; *p* < 0.001; *n* = 3345] and so were their interaction [*F*_(20, 3281)_ = 37.802; *p* < 0.001; *n* = 3345]. The three-way interaction of gender, emotion category and display type was also significant [*F*_(20, 3281)_ = 2.184; *p* = 0.002; *n* = 3345].

### Japanese sample

#### Closed questions (Q2)

##### Descriptive analysis

**Figure 9:**
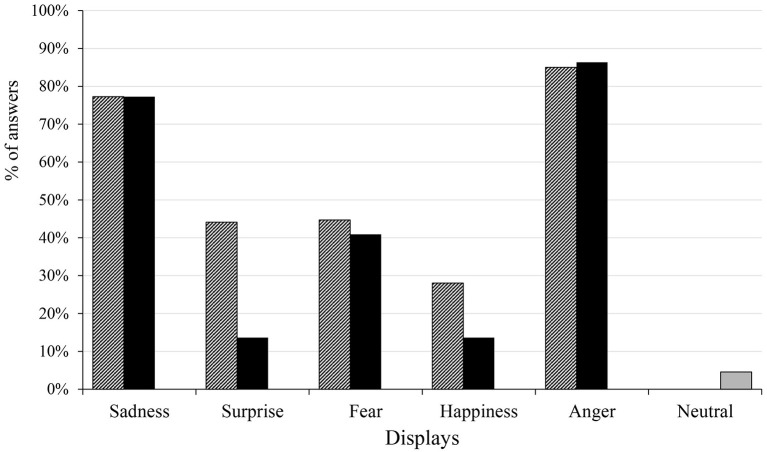
The striped columns show the largest score given to the correct emotions, the black columns show the maximum 5 score given for the correct emotions, while the gray column depicts the percentage of answers in which the participants gave 5 scores for any emotions in case of the neutral display in the Japanese group.

**Figure 10:**
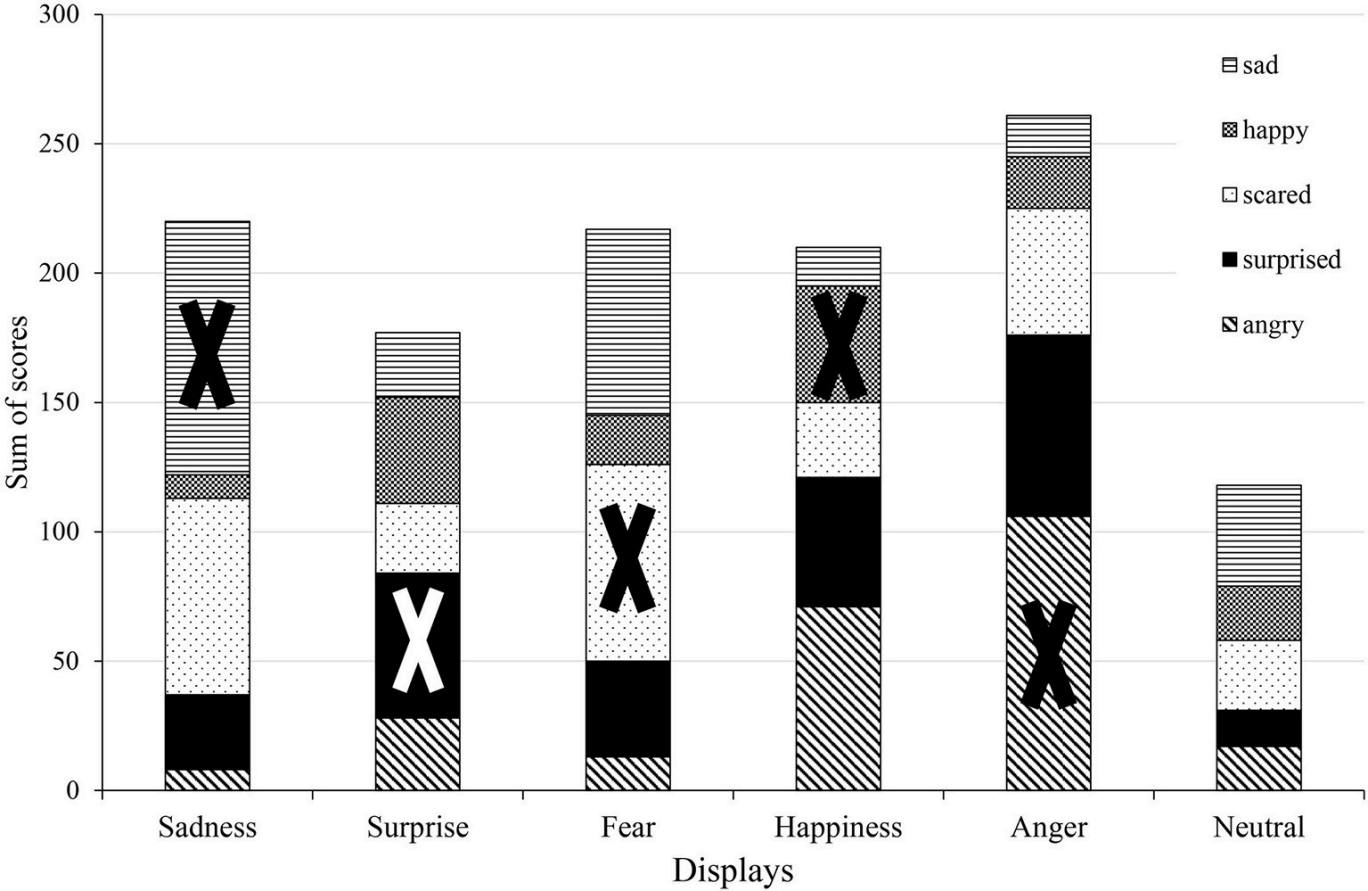
Analysis of sum of scores in the Japanese group. The X sign shows the correct emotions in each display.

The participants gave the biggest score to the correct emotion more than half of the time in the Sadness (77%) and the Anger (85%) displays, and these scores were usually the maximum 5 score which indicates the participants' high certainty when scoring the correct emotions. The percentage of answers were close to 50% for the largest value in case of the Surprise (44%) and the Fear (45%) displays, but in the Happiness display it only reached 28%. Both in the Surprise and Happiness displays the certainty was fairly low, as only 14% of the answers gave the maximum 5 score. The neutral display has rarely received a maximum score for any emotions (5%), reassuring that the participants did not strongly associate it with the five emotions (Figure 9). The analysis of sum of scores is visualized in Figure 10, showing that the Anger display received the most scores, while the neutral state received the less. Together with the confusion matrix, the results show that the sad and afraid emotions were mixed frequently in the Fear display (afraid: 47%, sad: 35%) and in the Surprise display surprised (46%) was sometimes mixed with the happy (32%) emotion. The most mismatched display was Happiness, in which only 28% of the answers gave the biggest score to happy, and most of the participants recognized it as angry (51%). The neutral display was most recognized as sad (40%), followed by afraid and happy, both only at 21%.

##### Statistical analysis

**Figure 11:**
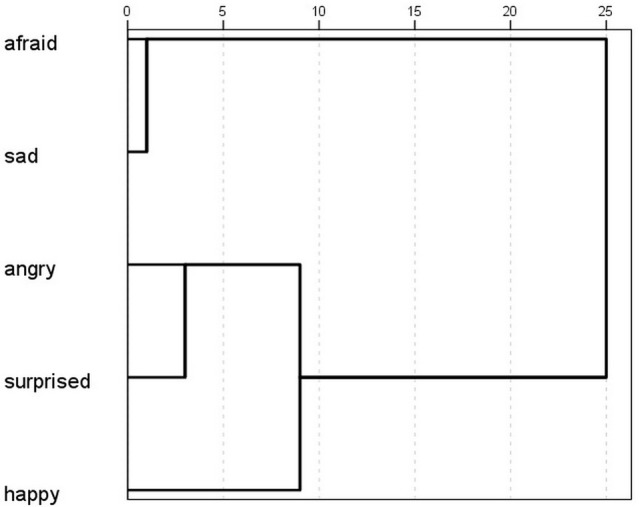
Ward's hierarchical cluster analysis showing the similarities of scores given to each emotion by the Japanese participants.

The success of recognition of the displayed emotions was significantly higher than chance level in most cases in the permissive success (in the sad, afraid, surprised and angry displays *p* < 0.001 while it was not significant in the happy display *p* = 0.056). In case of the strict success the recognition was significantly higher than chance level in the sad and angry displays (*p* < 0.001) but not in the surprised, afraid and happy displays (*p* = 0.133; *p* = 0.267; *p* = 0.457). We compared the scores given to the correct emotions to the scores given to the other emotions with Friedman tests and Wilcoxon signed-rank tests and found significant difference in the Sadness (sad-happy, sad-angry, sad-surprised: *p* < 0.001 sad-afraid: *p* = 0.007) and Anger displays (angry-afraid, angry-happy, angry-sad: *p* < 0.001 angry-surprised: *p* = 0.001) where the scores of the correct emotion differed from the rest. In case of the Surprise and Fear displays only one emotion did not differ in its scores from the correct one (surprised-happy in the Surprise display and afraid-sad in the Fear display) while the rest received significantly less scores than the correct answer. The scores of the correct emotion did not differ from any other in the Happiness display. Due to only having one female participant we did not include gender in the GLMM analysis. GLMM showed that the overall corrected model was significant [*F*_(29, 626)_ = 10.505; *p* < 0.001; *n* = 660], and so were the effects of display type [*F*_(5, 626)_ = 10.358; *p* < 0.001; *n* = 660], emotion category [*F*_(4, 626)_ = 10.402; *p* < 0.001; *n* = 660], and also their interaction [*F*_(20, 626)_ = 13.402; *p* < 0.001; *n* = 660]. The hierarchical cluster analysis showed that participants usually scored displays similarly for the afraid and sad emotions, indicating that if a display was scored as sad, it was likely also scored as afraid (Figure 11). Angry, surprised and happy were closer together, therefore in the Japanese sample we can see a similar pattern of scoring, as the high arousal (angry, surprised, happy) were more likely to be scored the same in the displays.

### Intercultural comparison

We used matched subject groups to provide an appropriate basis for the intercultural comparison of Hungarian and Japanese subjects. We excluded the only female participant from the Japanese group, and selected from the total Hungarian subject group a subsample matched based on gender and age to the Japanese subjects. The Japanese sample consisted of 21 male participants (age 22.3 ± SD 1.1 years), similarly to the matched Hungarian sample which consisted of 21 participants (age 21.1 ± SD 1.3 years).

#### Closed-ended questionnaire (Q2)

##### Descriptive analysis

**Figure 12:**
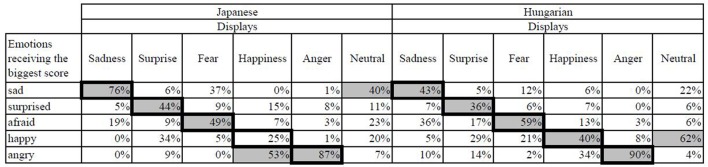
Confusion matrix for the answers of the matched Japanese and Hungarian participants. The gray cells indicate the percentage at which each emotion received the biggest score. The correct emotions are indicated by dark borders.

**Figure 13:**
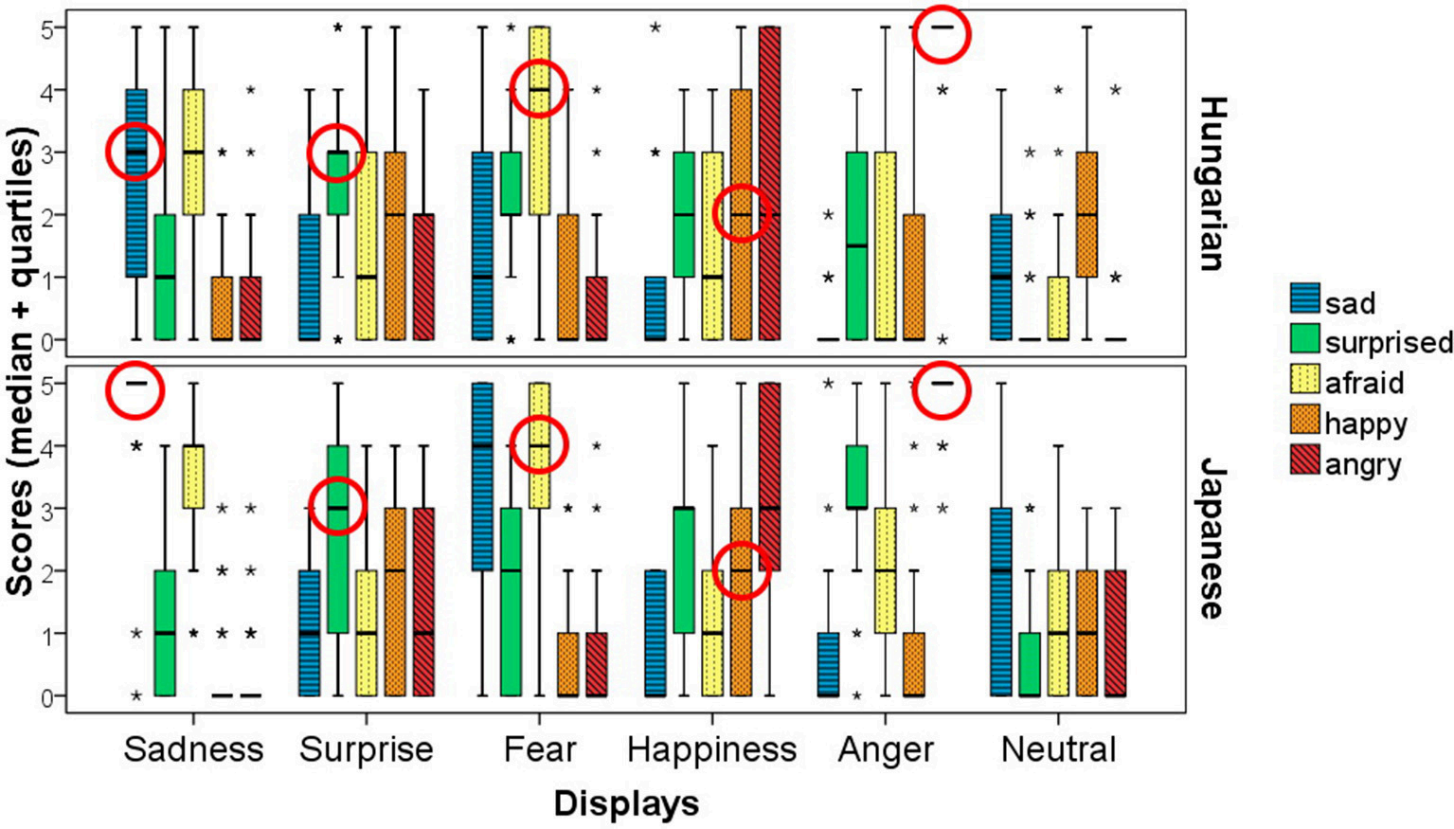
The figure shows the scores given for the six displays, while the circles show the medians for the scores given to the correct emotions by the Japanese participants and the participants in the matched Hungarian group. The * indicates outliers.

Based on the confusion matrix (Figure 12) and the scores given for the six displays (Figure 13) Japanese participants gave high scores in case of the Sadness display for the sad emotion, while Hungarians gave similar scores to both the sad and afraid emotions. The Surprise display received high scores in both groups with the biggest median for the surprise emotion, but the other emotions also received high scores. In case of Fear, Hungarians gave more scores to afraid, while Japanese participants gave similar scores to the afraid and sad emotions. The Happiness display was scored mainly as happy, angry or surprised in both groups, but in the Japanese group the scores given to surprised and angry exceeded the scores given to the happy emotion. The Anger display was overwhelmingly scored as angry by both groups, but surprised and afraid also received high scores. Neither of the emotions was given high scores in the Neutral display, but Hungarians considered the display to be somewhat happy, while Japanese participants scored it rather as sad.

##### Statistical analysis

In the GLMM analysis we did not include gender due to the all-male subject groups, but included nationality as a fixed effect. The overall corrected model was significant [*F*_(59, 1196)_ = 8.146; *p* < 0.001; *n* = 1260]. The effects of display type [*F*_(5, 1196)_ = 13.576; *p* < 0.001; *n* = 1260] and the emotion category [*F*_(4, 1196)_ = 8.215; *p* < 0.001; *n* = 1260] were significant, but not the effect of nationality, or its interaction with the display type. However, the nationality-emotion category interaction was significant [*F*_(4, 1196)_ = 5.451; *p* < 0.001; *n* = 1260], as well as the three-way interaction of display type, emotion category and nationality [*F*_(20, 1196)_ = 1.998; *p* = 0.006; *n* = 1260].

## Discussion

The first hypothesis concerned the correct recognition of emotions. The results show that in most cases the subjects could recognize the emotions that the agent's display was meant to convey in the closed-ended questionnaire. The Hungarian participants recognized the Anger display correctly based on both the descriptive and statistical analysis, and the certainty of their answers was the highest in this display as they gave the maximum score almost 80% of the time. The recognition of anger from human facial expressions is around 92% in adults in forced choice tests and 78% in a free-label test (Russell, 1994) and 88% in 5–7 years old children (Durand et al., 2007). In the eMOTO study by Fagerberg et al. (2004), some aspects of emotional expression showed similarities with our displays, as they also used more angular forms and strong red colors to express anger. These similarities might arise from the cultural use of colors, but that can also be based on the color's biological relevance, for example red is frequently found in warning colors (Stevens and Ruxton, 2011), or during the increase of facial blood flow that frequently co-occurs with anger, which leads to redness in the human face (Drummond and Quah, 2001). The strong interplay of cultural and biological expressions of anger might have contributed to the high recognition of this emotion in our agent. Although the recognition of the other emotions did not reach the usual recognition rates of facial displays of emotions (Russell, 1994; Elfenbein and Ambady, 2002), the displays were usually recognized above chance level. The Neutral display proved to be perceived as truly neutral as it did not receive high scores for any emotions. In studies examining emotions expressed by social robots the recognition rates are similar or lower than that of human facial expressions, e.g., Probo: 100–65% (Saldien et al., 2010); Kismet: 86–57% (Breazeal, 2003); Eddie: 90–20% (Gonsior et al., 2012). Although some features of these robots are animal-like or stylized, their emotion expressions are mostly based on human facial expressions. Emotions expressed via virtual faces are recognized between 97.86% (happiness) and 22.96% (disgust) (Dyck et al., 2008). In a study by Gácsi et al. (2016) the multimodal emotion expressions of a social robot were based on emotionally expressive behaviors of dogs instead of human facial expressions. The percentage of correct answers were slightly higher than in our current study (88–62%), which can be due to the physical embodiment of the robot.

In the open-ended questionnaire the answers depicting the correct emotions differed widely by display, ranging from 16% (Sadness) to 64% (Anger). These percentages are much lower than the success of recognition achieved in the closed-ended questionnaire. Which is to be expected, considering that the participants had no prior experience with similar emotion expression agents, and the open-ended format enabled them to also answer with words depicting different emotions or states than in the closed-ended format.

Following our second hypothesis, the lower rate of recognition can be explained by the similarities of behavioral attributes between some of the displays. In the open-ended questionnaire some inner states, like tired, sleepy or thinking were mentioned in the case of several displays, but their prevalence was the highest in the Sadness display. As sadness is a low arousal emotion (Russell, 1980) it can be difficult to differentiate between inner states like tiredness or sleepiness and the expression of sadness on the behavioral level, even in the case of humans (Nettle, 2009), especially if there is no additional context. The answers were the most divided in case of the Surprise display, which can originate from the lack of clear positive or negative valence, as the reaction to sudden stimuli usually depends on the situation and the stimulus itself (Russell and Barrett, 1999), making the recognition more difficult without a clarifying context. In the closed-ended questionnaire the Fear and Sadness displays were recognized correctly to a similar extent in the Hungarian sample, 53 and 59% of the answers. While the statistical analysis showed that the scores given to the correct emotion differed significantly from the rest in the Fear display, for the Sadness display there was no significant difference between scores of the afraid and sad emotions. The mixture of the afraid and sad emotions was more prevalent in this display, but it was present in the case of the Fear display too, to a lesser extent. The mismatch between these two emotion displays could be the result of their similarly negative valence and some of the shared actions of the displays (pale color, size decrease). Similarly, the other prevalent mismatch was observed in the case of the Happiness display, in which participants also gave high scores to the angry emotion. This confusion might come from the similarities of intensity levels in the happy-angry emotions which are both high arousal emotions, and show some similar behavioral elements such as fast movement, perceived increase in size and vivid colors. Surprise is also a high-arousal emotion, which is reflected in its closeness to the angry and afraid scores in the cluster analysis. This reinforces the idea that the arousal level was a decisive factor for the scores given by the participants. The low certainty of the scores given for the surprise display can be the result of the ambiguousness of the emotion, as it has neither inherently positive nor negative valence.

When we look at cultural differences we can see that in most cases the participants from the two cultures recognized the displays similarly, but we also found differences. Hungarian and Japanese participants considered the neutral state to have a slightly different valence: while Hungarians scored it as more happy, Japanese participants perceived it to be somewhat sad. Additionally, the confusion matrix shows that Japanese participants were more successful in the recognition of Sadness than Hungarians, and they also gave high scores for sadness in the Fear display. As the human fine tuning step (see preliminary study in section Procedure) was conducted with Hungarians, it is possible that parameters such as the color values of the agent in these displays (mostly light blue or fading) or the rotation speed was considered low arousal by Japanese participants. The Anger and Surprise displays received similar scores in the two groups, reflecting the results already found in case of the full Hungarian subject group. Japanese participants were less successful in the recognition of Happiness than Hungarians, and they also tended to interpret it as more angry. The Happiness display of the agent was developed to express a high intensity emotion, but in many Asian cultures happiness is associated with a more relaxed, less intensive experience (Tsai et al., 2006; Miyamoto and Ma, 2011). Although the differences show the need for cultural fine tuning, the majority of displays received the highest scores for the correct emotions, indicating that the general rules used for creating the emotion expressions can be considered a good basis for emotion expression in artificial agents.

### Limitations

Although our results prove that using the biological rules for creating the expressions was generally successful, our study has some limitations which has to be considered. As we only wanted to test the concept, we only used one agent to which we created the emotion expressions, and all subjects saw the exact same emotion expression displays. These two aspects can lead to pseudoreplication effects (Heffner et al., 1996), which could have been an issue in case of a different research question, one that would set out to measure the efficiency of these displays in detail, and compare them to more convenient emotion expressions. In contrast, pseudoreplication is not a major issue in this current research, as we focused on testing the applicability of the theory. In the future we intend to investigate the implemented concept on various, broadly different artificial agents. We plan to embed a randomization effect which will generate slight variations within the set parameter limits for each emotion, providing variable displays that will also ensure the avoidance of pseudoreplication.

In the study we acquired data from both Hungarian and Japanese participants, which gave us the opportunity to conduct intercultural comparisons. Although we used matched subject groups for direct comparisons, these groups consisted of considerably less participants than the total Hungarian sample, and included only male participants; therefore the intercultural aspect of our study only opens new opportunities for more detailed cross-cultural research.

### Ethical considerations

The emotion expression of artificial agents, especially in social robotics and ICT is becoming an increasingly researched area and can potentially lead to more diverse and robust human-machine interactions. Artificial agents are becoming more pervasive in our day-to-day life, and are expected to become even more widespread in the near future. With the growth of social robotics and the immersion of mobile applications in our daily tasks, ethical questions that seemed theoretical a few decades ago are of practical value today. It is expected that social robots will be used in e.g., elderly care, education, households and healthcare, in which these agents will interact with various people who are not familiar with the capabilities and limitations of robots. Communication technologies and especially mobile devices are already owned by the majority of the population in western countries (Donner, 2008) and have become the integral part of our culture and the way we interact with others (Srivastava, 2005). In case of long-term interactions with social robots or use of communication devices, people may attribute higher, anthropomorphic skills and cognitive features to these agents than what they really possess (Miklósi and Gácsi, 2012). A big emphasis should be put on truthful informing about agents that show behaviors associated with higher cognitive functions. It is known that people can get attached to their smart phones (Konok et al., 2016) and in certain areas social robotics is also encouraged to develop robots whose behavior facilitate the forming of attachment between the human and the robot in long-term interactions (Miklósi and Gácsi, 2012). The development of emotion expression for artificial agents requires a cautious approach which ensures that the new technologies that choose to integrate any kind of emotion expression in their devices could not be used to mislead or exploit users, especially the ones that generally have more difficulties understanding the concepts and limitations of these devices. We strongly believe that by developing less anthropomorphic emotion expressions, and by only using them within their constraints, this approach could be favorable. We plan to conduct research on the effects of any emotion expression in artificial agents, and encourage other researchers to do likewise.

## Outlook

Using general biological rules to create simple expressive behaviors for artificial agents proved to be a correct approach. We wish to establish the concept further by implementing and examining the biological approach in various agents with a diverse set of features and functions, both in connection with ICT applications and social robotics. In the current agent we did not implement disgust, as it is defined more as a sensory affect instead of an emotion (Panksepp, 2007), but it might be included in future studies. The development of new agents should also focus on the individualization of the agents. This could be achieved with the innovation of the agent's appearance and structure, while fine tuning of the agent (e.g., creating long-term personalities or short-term moods) would be possible by adjusting the display of the neutral state or the intensity of emotional displays. The development of continuous, adequately dynamic emotion expression is the aim of future studies.

## Data availability statement

Datasets are available on request; The raw data supporting the conclusions of this manuscript will be made available by the authors, without undue reservation, to any qualified researcher.

## Author contributions

MG, ÁM, TF, PK, and PB developed the main concept, design and the new approach of the study. MG, TF, and ÁM developed the ethological basis of the emotion expressions. GP implemented them as visual displays. MG, MN, and TF tested the participants with the questionnaires. BK, MG, VK, and TF conducted the statistical analysis. BK, VK, MG, ÁM, and TF wrote the manuscript. All authors contributed to the manuscript and approved the final version.

### Conflict of interest statement

The authors declare that the research was conducted in the absence of any commercial or financial relationships that could be construed as a potential conflict of interest.
